# Rethinking authentic assessment: work, well-being, and society

**DOI:** 10.1007/s10734-022-00822-y

**Published:** 2022-02-17

**Authors:** Jan McArthur

**Affiliations:** Department of Educational Research, Lancaster University, Lancaster, UK

**Keywords:** Authentic assessment, Well-being, Critical theory, Transformative change, World of work

## Abstract

This article seeks a deeper understanding of the concept of authentic assessment which ensures it does not become another educational buzzword, slowly diminishing in real meaning. I consider the origins of the term in the US schooling sector, and how it has developed over time, and in different countries, to today focus in higher education largely on real world tasks. There is, however, I argue, a common conflation of real world with the world of work. Little of this literature actually engages with the rich philosophical debates on authenticity, and in this article, I suggest that this deeper understanding of authenticity can enable us to build on existing work on authentic assessment to develop a more holistic and richer concept that will be more beneficial to individual students and to the larger society of which they are part. I argue that we should move from thinking in terms of either the so-called real world, or the world of work, to focus our justification for authentic assessment on its social value (which encompasses but is not limited to its economic value). To achieve this aim, I suggest we move from simply focusing on the authentic task to considering why that task matters? This then enables a shift from the student in isolation to the student as a member of society. Senses of achievement can become richer, thus enhancing the students’ sense of self, self-worth, and well-being.

## Introduction

For academic activity in higher education to be self-perpetuating it has to some extent to be unworldly. In each generation higher education has to provide exemplars for the next generation of socially uncommitted and irrelevant scholarship, teaching, and research. For only by preserving this element - which does not mean in practice preserving it in every single corner of higher education but generously spreading it through-out - can it begin to maintain capacities for neutrality and independence, for objectivity and disinterestedness which may make what it has to offer at the same time socially useful. Too close a social involvement can make higher education socially useless (1975, p. 427).

The contrast between Weinstein’s, 1975 vision of higher education (in this journal) and prevailing attitudes today is stark. Where Weinstein valorises separation from broader society, today universities around the world are expected to have explicit, close, and direct links to wider society, and particularly the economic realm. One important way in which contemporary higher education has promoted its relationship with the wider world is through the concept of authentic assessment, which is variously understood as assessment linked to real-world tasks, the world of work, or authentic knowledge. The increasing international focus on the idea of authentic assessment demonstrates the importance of reflecting deeply on what we do and do not wish to achieve through this form of assessment. Authentic assessment in higher education has been particularly prominent in the UK (Maclellan, 2001; Rust, 2007; Sambell & Brown, 2021; Sambell et al., 2013) and Australia (Ajjawi et al., 2019; Ashford-Rowe et al., 2014; Herrington, 2014; Herrington & Herrington, 1998); however, we can also see engagement in other parts of the world: for example, Chile (Villarroel et al., 2018), Indonesia (Arlianty et al., 2017; Sutadji et al., 2021), Sri Lanka (Karunanayaka & Naidu, 2021), Singapore (Chong et al., 2016), Netherlands (Gulikers et al., 2004), South Africa (Maniram & Maistry, 2018), and Botswana (Oladele, 2011). Interest in authentic assessment also covers many disciplinary fields, including those in STEM, health, and social sciences.

Much of the authentic assessment literature uses a fairly disarticulated and ahistorical sense of authenticity, with little or no reference to the significant philosophical discussions of the term. The most common understanding of authentic assessment in higher education describes it as involving ‘real-world’ tasks (Ashford-Rowe et al., 2014; Bryan & Clegg, 2006; Karunanayaka & Naidu, 2021; Rust, 2007). There is also a frequent assumption that this ‘real world’ is the world of work: ways of applying knowledge and skills that graduates would use in the professions and workplace (Ashford-Rowe et al., 2014; James & Casidy, 2018; Karunanayaka & Naidu, 2021; Villarroel et al., 2018; Wiewiora & Kowalkiewicz, 2019). It is this conflation of the real world and the world of work which I find troubling (McArthur, 2020). This lack of deeper philosophical understanding of the idea of authenticity risks it becoming something of a buzzword, as happens too often in higher education. In an online blog ahead of a conference, I recently warned:

Higher education is also adept at taking a good idea and turning it into a buzzword, from which the original meaning drains away. A buzzword concept is no longer reflected on, challenged or probed. It becomes so obviously what we must do, that we no longer contemplate why we should do it. (McArthur, 2021)

Indeed, in 1964 Adorno (2003) warned of the ‘jargon’ of authenticity:

Whoever is versed in the jargon does not have to say what he thinks, does not even have to think it properly. The jargon takes over this task and devalues thought. (6)

I should stress, however, that the existing scholarship on authentic assessment is rich and most is genuinely directed to students’ best interests (e.g. Ajjawi et al., 2019; Ashford-Rowe et al., 2014; Forsyth & Evans, 2019; Herrington & Herrington, 1998). My exploration of authentic assessment is built on this earlier work. But Adorno’s quote does give a salutary warning. Policy makers and senior management in higher education love to grasp buzzwords. Before we know it, authenticity will be mandated in every assessment—and its meaning will be lost.

It is therefore fair to observe that, apart from general references to the real world or world of work, much of the literature on authentic assessment does not critically consider what the term authentic might mean (e.g. Bearman et al., 2017; Jackson & Bridgstock, 2021). Indeed, the promotion of authentic assessment is generally linked to innovative approaches to higher education teaching, learning, and assessment (Sambell & Brown, 2021; Sambell & McDowell, 1998). This is important work, but it is not directed at broader social and political change in this context of authentic assessment. A rare exception is the work of Forsyth and Evans (2019) which seeks to use authentic assessment to address decolonialisation and the need for more inclusive approaches to teaching history.

Consider, as a comparison, the expansive way in which the term authentic is used when we write about ourselves: our profession as academics and teachers or the idea of authentic academic life. It is very often far richer than simply ‘real world tasks’. For example:

The results revealed that authenticity in teaching consisted of themes of being one’s own self, pedagogical relationships, contestation, and ultimate meaning which were enacted in the participants’ practices through their sense of responsibility, awareness of their possibilities, understanding of pedagogical relationships, self-reflection, critical reflection, and critical hope. (Ramezandadeh et al., 2017, p. 299)

Such an understanding of authentic should extend to our students and their achievements. The idea of authentic assessment that I develop in this article seeks to make three important shifts in our conceptual understanding and practical application: firstly, to shift our focus from the real world/world of work to a richer understanding of society as a whole; secondly, to transcend a focus simply on the task, and consider instead the value of that task; and finally, I outline an understanding of authentic assessment which does not reinforce the status-quo but is instead a vehicle for transformative social change.

The article is in five parts. After this introduction, I give a brief history of the term authentic assessment, highlighting its origins in US work on alternatives to standardised testing in schools, and how the term has expanded internationally and into different meanings. In order to ground authenticity in a stronger philosophical base, I then consider the very different viewpoints of Heidegger and Adorno. The main section explores how we can rethink the rationale for authentic assessment: why we should embrace it in higher education, but also in what form. I conclude with a brief discussion of the implications of adopting this revised understanding of authentic assessment.

## A brief history of authentic assessment

Early use of the term authentic assessment comes from the USA at the start of the 1990s. There is little mention of so-called real-world tasks; instead the authenticity is conceived on the basis of the knowledge being evaluated. Wiggins (1990) describes authentic assessment in terms of a direct assessment of what the student is meant to have learned, which therefore does not rely on common proxies for evaluation. Such proxy forms of assessment get the student to do something a bit like a real task and then make inferences about their achievement from that. Wiggins and others writing at this time were responding to standardised testing—which was a cornerstone of the US educational system. This early literature promoted the educational merits of a more time intensive, but richer, approach to assessment within schools.

Through the early 1990s, literature on authentic assessment continues to have a focus in US secondary education, often through a psychological lens. A small sign of the future development of the concept comes in Wooster’s (1993) article linking authentic assessment to global education. One of the earliest references to authentic assessment being about real-world problems or tasks can be found in an account of a teaching innovation led by educational researchers in Massachusetts (McFarlane, 1993). But the emphasis remains largely on better ways to assess work, rather than on outside tasks or roles. Common among early references to authentic assessment is the mention of portfolio assessment—gathering together a range of students’ best work in order to evaluate progress. Some evidence of the concept crossing the Atlantic can be found in Torrance’s (1993) analysis of links between the ‘American idea’ of authentic assessment and the national school curriculum in England and Wales.

Australian, John Biggs (1996), while working in Hong Kong, is influential in bringing the concept of authentic assessment into higher education in an early outline of constructive alignment (in this journal). However influential, Biggs’ meaning of authentic remains somewhat under-developed at this stage. An early example of the direct link between higher education, authentic activities, and the workplace comes in Seagraves et al. (1996) study of the relevance of academic outcomes to the workplace (again in this journal).

Another early example of authentic assessment crossing into the higher education literature comes from medical education (Snadden & Thomas, 1998). Here again authentic assessment is very much associated with the use of portfolios and, in this case, evidencing the application of theory in practice. In a similar way with reference to teacher education, Riley and Stern (1998) make an argument for authentic assessment bridging theory and practice. They criticise the conflation of authentic assessment with both alternative assessments and performance-based (reflecting, however, common usage in the US literature) and instead refer to Thermer’s work of 1996 in which the concept is understood in terms of describing ‘the process of evaluating a learner’s original piece of work or completed task, arrived at based upon a previously acquired body of knowledge, and demonstrated in a concrete form’ (178).

To return briefly to the US school literature on authentic assessment, we should note that its broader focus on the nature of the task and knowledge continued even as higher education’s use of authentic assessment began to focus increasingly on the world of work. For example, Newmann, King, and Carmichael (2007) focus on early years of schooling as well as later years. Their emphasis is therefore on assessment that has value and relevance beyond the classroom or school, but not necessarily work. For an 8-year-old student, it may not mean a lot to think about their working life at least a decade away, but the application of knowledge in the playground or at home offers something more than just learning something in the classroom for the purpose of being tested in the classroom.

### From alternative assessments to real-world tasks

By the late 1990s, authentic assessment is appearing regularly in the mainstream higher education literature in many western countries, and a few years later, also in literature from many other parts of the world (e.g. Arlianty et al., 2017; Chong et al., 2016; Gulikers et al., 2004; Karunanayaka & Naidu, 2021; Maniram & Maistry, 2018; Sutadji et al., 2021; Villarroel et al., 2018). Early use in the mainstream higher education and assessment literature appears to follow the association of authentic assessment with alternative forms of assessment (e.g. Sambell & McDowell, 1998), even though this is simultaneously challenged in the teacher education literature, as outlined above, by Riley and Stern (1998). Often such alternative/authentic forms of assessment involved reflective journals and portfolios (e.g. Woodward, 1998).

At this time, Joughin (1998) gives a clear definition of authentic assessment in higher education, which seems very much in line with our current usage today: ‘”Authenticity”, he argues, refers to the extent to which assessment replicates the context of professional practice or “real life”’ (371). But the association with alternative assessments—alternative generally meaning in comparison to traditional exams and pen and paper exercises—continues for some time (e.g. Maclellan, 2004).

An important work by Herrington and Herrington (1998) connects authentic assessment to work on situated learning which began to emerge a decade earlier. This ground-breaking work on the social nature of learning, such as from Brown et al. (1989), challenges traditional psychological and purely cognitive approaches to individual learning. It is an important step for the argument I aim to construct here, whereby connecting work, well-being, and society, the link to social and situated forms of learning is essential. When learning itself ceases to be understood purely as an individual act of cognition, our ways of assessing that learning should also necessarily change.

In 2003, Tynjälä et al. (2003) observed in this journal that the relationship between higher education and its environment had profoundly changed over the past two decades. Indeed, much about the quote from Weinstein in 1975, at the start of this article, is already looking outdated. And yet, this 2003 perspective supports just the same worry Weinstein appeared to have in 1975, namely that moving from an elite to a mass system would change the relationship between higher education and the broader realm in which it is located.

Another way of extending the perspective of authentic assessment comes with Boud and Falchikov (2006) who connect it to their work on assessment and longer-term learning. While generally supportive of the idea of authentic assessment, they warn that it does not on its own, or necessarily, support the longer-term ambitions of learning in the future on which they focus. Elsewhere these authors do not explicitly link authentic assessment and work, but rather acknowledge the wisdom of authentic assessment approaches that connect the knowledge learned to how that knowledge may be later applied (Boud & Falchikov, 2007). Kearney (2013) also brings together sustainable (life-long) assessment, authentic assessment, and peer and self-assessment for learning.

Evidence of the cultural shift in our thinking of assessment comes from Bryan and Clegg (2006) who argue that by the mid-2000s the evidence base for innovative forms of assessment is well established. Their understanding includes the idea of authentic assessment, understood as actively participating in real-world tasks that enable the application of knowledge and skills. So, we see innovative and real-world being used almost as synonyms. Rust (2007) then builds on this as he seeks to establish a scholarship of assessment, and includes active engagement with real-world tasks as one of the features of any scholarship of assessment.

Trevitt and Stocks (2012) provide an interesting approach to authentic assessment by exploring the concept of authenticity through the work of educational theorists such as Carolin Kreber and Jon Nixon, including notions of being self-aware and true to oneself. Thus, they do move closer to a philosophical understanding, and yet Trevitt and Stocks also pull this back into a largely work-based context of professional learning. One of the few specifically philosophical accounts comes for Vu and Dall’Alba (2014) where they use Heidegger’s concept of authenticity to explore assessment.

In 2014, Ashford-Rowe et al. (2014) sought to identify the key aspects of authentic assessment. By synthesising the rich literature in this field, the authors propose eight critical elements of authentic assessment. These are:

Authentic assessment should be challenging

The outcome of an authentic assessment should be in the form of a performance or product (outcome)

Authentic assessment design should ensure transfer of knowledge

Metacognition as a component of authentic assessment

The importance of a requirement to ensure accuracy in assessment performance

The role of the assessment environment and the tools used to deliver the assessment task

The importance of formally designing in an opportunity to discuss and provide feedback

The value of collaboration (pages 207–210)

As they appear here in this list, these critical elements seem to reasonably capture many dimensions of authentic assessment; however, as each is elaborated in the larger text, very quickly several elements are explained and justified by the narrower reference to work or the workplace. This highly influential overview of authentic assessment itself falls into this problem of a conflation of world of work with real world. It is, however, clear that many authors who reference authentic assessment in terms of the workplace are not necessarily intending this as a passive understanding of the workplace (e.g. Villarroel et al., 2018). And it is also the case that because their focus is on the acts of assessment, some of the broader issues of how economic and social factors inter-relate in society are not at the fore of their analysis, and nor are they intended to be.

It strikes me as problematic, however, to suggest, as Wiewiora and Kowalkiewicz (2019) do, that this work/real world focus necessitates we have too much focus on knowledge in higher education and instead need to foreground skills and competencies. A more appropriate approach is the way in which Ajjawi et al. (2019) bring authentic assessment specifically into the realm of vocational education. This issue of skills or knowledge is important and has had some, but as yet insufficient, attention in the literature on authentic assessment. A welcome development can be found in the impressive range of examples of authentic assessment compiled by Sambell and Brown (2021). These suggest a return (or continuation) of Sambell and McDowell’s (1998) earlier view of authentic in terms of alternative forms of assessment. Sambell and Brown focus particularly on alternatives to the traditional essay and in this sense helpfully transcend the limitations of a real world/world of work approach that often appears to exclude non-vocational subjects from the realm of authentic assessment. In contrast, the systematic review of literature on authentic assessment by Sokhanvar, Salehi, and Sokhanvar (2021) has the narrower focus of links with employability as does Sotiriadou et al. (2020).

I turn now to what we may gain by greater engagement with the philosophical literature on authenticity, in order to re-imagine authentic assessment. Here I focus specifically on Heidegger and Adorno, as the juxtaposition of their philosophies provides a helpful frame to my own critical theory approach (using Adorno) and existing work on authentic assessment using Heidegger (see Vu & Dall'Alba, 2014).

## Authenticity: the significance of Heidegger and Adorno

Authenticity is a vexing concept. It promises so much and yet is so easily devalued, as in marketing campaigns for authentic travel experiences and the like. I believe we can build a stronger, more transformative, idea of authentic assessment by building on the existing scholarship through the introduction of more reflection on the meaning of authenticity. Heidegger is a towering figure in the literature on authenticity, and his influence is undeniable. And yet, as a critical theorist, I am sympathetic to Adorno’s (2003) critique of Heidegger, and explaining why will establish the foundations for my later discussion of an alternative understanding of authentic assessment.

Heidegger’s (1962) authenticity is about our relationship to self or being. There is therefore no *one* authenticity as it is unique to each person’s relationship to self. One cannot understand Heidegger’s authenticity from the outside: it is ‘only relevant to the individual and their relationship with their being’ (Trubody, 2015, p. 22). This intensive focus on self is fundamental to the existential tradition in which Heidegger’s work is typically located (Blackburn, 2016). Conceptually, this leads to a number of problems if we try to use this as a foundation for authentic assessment. The idea of authenticity solely as a relationship to self makes it problematic to use the term in any sort of systematised way: hence, the idea of an authentic curriculum or authentic education ‘seems completely removed from Heidegger’s sense’ (Trubody, 2015, p. 22). As Trubody argues, many who use Heidegger to ground their idea of authenticity do so in ways probably quite remote from what Heidegger actually meant.

In *The Jargon of Authenticity*, Adorno (2003) offers a damning and visceral critique of Heidegger’s work. Reading Adorno’s text, it is clear that he has no issue with the word authenticity itself (an observation confrimed by Jay, 2010) but rather with what he regards as the unhistorical, selective, and illusionary way Heidegger makes use of it. To understand his objections, one must understand the key differences between an existential philosophy and critical theory. As a critical theorist, Adorno’s understanding of the self and individual is definitively different to that of an existential philosopher. For critical theory, ranging from Horkheimer’s (1993) early elucidation to Honneth’s (2004b) contemporary work, the fundamental idea is the inter-relationship between self and society in a paradoxical sense that requires both cooperation and autonomy. Heidegger’s self is about escaping bonds; critical theory is about being nurtured (or harmed) by relationships. While Heidegger places the self in an historical context to some extent, it differs significantly from the immanence at the heart of critical theory: that basis in history and the world as it is, while simultaneously seeking to transcend that world (Fraser, 2003). It is these features at the heart of critical theory, which I bring to my rethinking of authentic assessment.

Heidegger is clear that because authenticity is about self, authenticity can only ever be known by that self, and every instance of authenticity is different, and thus there can be no general sense of what it is. For some, such as Macdonald (2008), this seems very close to Adorno’s central idea of non-identity. Macdonald and Ziarek (2008) even suggest that the conflict between Adorno and Heidegger is sharpened because of ‘undeniable points of proximity’ (2): they seem to imply that Adorno is cross because Heidegger has wandered into his territory. Putting Adorno’s motivations aside, what these authors miss is how Adorno’s non-identity rests in a context with a belief in immanent understandings of our situation and the associated strong inter-relationship between self and society. Thus when Trubody (2015) argues that Heidegger’s sense of authenticity cannot be systematised, the enormity of the gulf between it and Adorno becomes clear. Adorno (1973) argues that attempts to tie objects into tidy definitions reflects our impulse to dominate nature, one of the most problematic legacies of the Enlightenment (see also Horkheimer & Adorno, 1997). Attempts to tie universal meanings to particular objects are always problematic because there is always something of the individual that is not reflected in the universal. While Adorno’s non-identity means there is an unresolvable limitation to our understanding of the world we are in, yet openly acknowledging this then allows for some consideration of historically situated change. From Adorno’s perspective, Heidegger does attempt to reconcile this inescapable state of unknowability. In a ‘slight of hand’, Heidegger makes his analysis appear to do something it simply does not do (Foster, 2007). Because in the end, Heidegger, according to Adorno and many critical theorists, does seek a ‘gold standard’ for authenticity (Jay, 2010): in other words, a definition.

Heidegger’s existentialism lacks the potential for transformative change inherent in a socio-historical perspective. That positive interdependence between individual and social well-being has powerful potential in terms of how change can happen. Thus, Heidegger’s authenticity may promise change, but in fact reinforces the status-quo (Swer, 2019). Grounded on self alone so that it cannot be systematised, as Trubody (2015) argues, there is little scope for it to form a conceptual foundation for educational or social change. This, for me, is the problem with earlier attempts to ground authentic assessment in Heideggerian existentialism, such as Vu and Dall’Alba (2014). They either need to misuse Heidegger’s key concept or misunderstand it.

## Rethinking authentic assessment: three key principles

Hanafin et al. (2007) offer one of the earliest suggestions that we could mean more by the concept of authentic assessment in higher education than real-world tasks or the world of work. Their argument that authentic assessment offers one of the best strategies for more inclusive forms of assessment, particularly for students with disabilities, thereby makes the connection to social justice. The implication of their analysis is that authentic assessment is not simply about the task, but the relationship the person/student has to the task and their sense of well-being.

Some time, later Forsyth and Evans (2019) also imagine authentic assessment in broader terms and explicitly relate it to challenging institutional norms, taken for granted practices and the hegemonic status-quo: very much at the heart of critical theory. Rather than adopting the jargon or buzzword of authentic assessment they ask the difficult question—‘Whose authenticity is it?’ In recognition of the decolonial movement and the imperative to recognise Indigenous epistemologies, they associate authentic assessment with challenges to homogenous and dominant epistemological assumptions and practices in how we assess knowledge.

Decolonialisation is but one of the urgent problems now facing higher education and wider society. In addition, we face the ever-growing climate emergency, a breakdown in civic trust, and the continued rise of groups and movements which seek to promote their interests largely through the denigration of the interests of others. Thus, to the extent that we ever talk about the real world, this is the world we should think about more in higher education: a world desperately in need of change. It should be referred to directly and explicitly and not through hopeful connections or wishful assumptions. It is on this basis that we take the existing work on authentic assessment and frame it beyond the real world as it exists or a narrow conception of work, and enable assessment to fulfil its role shaping how, what, and why our graduates gain the education that they will take forward into a range of social roles.

I offer three principles on which the idea of authentic assessment can be re-thought, with a stronger philosophical/conceptual foundation. I reject Heidegger’s authenticity of the existential self, and instead embrace the dialectic of self and society at the heart of critical theory, and a commitment to genuine transformative change. Firstly, I argue that we move from thinking of the real world or world of work to thinking in terms of society. Secondly, that we move from a focus on the task, to the reason for doing the task and how that validates the social belonging of the student. Finally, that we embrace a transformative relationship with society, with authentic assessment not just perpetuating what already exists, but propelling us on to a better future for all.

### From real world/world of work to society

As previously discussed, much of the higher education literature considers authentic assessment as real-world tasks and appears to conflate this with the world of work. This does not necessarily mean a lack of concern for student success and well-being (e.g. Ajjawi et al., 2019; Ashford-Rowe et al., 2014). But philosophically I think there are three problems with this conflation, which is why I suggest we should move to thinking of authentic assessment and society and neither the real world nor the world of work. First, there is a potential risk in conflating the real world with the world of work in that this can lead to a further hidden conflation such that this becomes simply what employers want. It can also be associated with a commodification of graduates’ identities such that they associate their self-worth with the exchange value of their labour. The pernicious idea of ‘graduate premiums’ (Ashwin, 2020) is a prime example of this. It suggests that the higher a graduate income, the more successful the student. What they do becomes alienated from who they are: value and worth are based only in the exchange value of their labour, and not what they do or what they contribute to society.

Neoliberal proponents of an unfettered market economy, such as Milton Freidman, would not see a problem in the logic of graduate premiums, because if the market pays someone a higher salary, it must mean the market judges them to be of higher worth. That is, in their world, the beauty of the market. But most of us know this is not true. We live in a mythic meritocracy (Frank, 2016; Littler, 2017; Liu, 2011; van Dijk et al., 2020). Indeed long forgotten is the fact that when Michael Young first used the term meritocracy in 1958 (Young, 2017), his use was satirical (van Dijk et al., 2020) and equally an argument against the idea as for. As the recent global pandemic showed, those most needed to ensure the well-being of the most people in society did not have salaries to match their ‘essential’ status. In this and many other ways, the pandemic has reinforced the illusionary nature of merit in many western societies (Sandel, 2020). I therefore argue that there must be something that we do in work as an activity that goes beyond what we are paid for that work, and this is what authentic assessment should instil in our students (note: this does not mean they should not be properly financially rewarded for their labour as well). This echoes Honneth’s (2004a) element of esteem recognition, in his plural understanding of social justice, and the importance to an individual of having traits or abilities through which they make a social contribution for which they are recognised.

The second problem is that this awful phrase ‘the real world’ makes it sound external to students and ourselves. The real world is *out there*, reinforcing a stereotypical image of the ‘ivory tower’ university which fails to recognise the university’s earliest foundations in labour, such as in the early craft guilds (Kivinen & Poikus, 2006). Indeed it is ultimately self-defeating to the notion of authentic assessment to position the real world as something out there and fundamentally separate from higher education. To do so, also has echoes of the now surely outdated opening quote from Weinstein?

Finally, portrayed in this rarified way, the real world becomes something that we cannot change. Students must accept it as given. Even in the case of those who link authentic assessment and sustainable assessment (Boud & Falchikov, 2006), there is very little sense that this long-term future the student is preparing for is something that they can shape or influence.

The best way to avoid all these traps is to positively re-embrace the concept of society, for so long made unpopular by neoliberals and radical individualism—and some might argue Heidegger’s existentialism (and often also linked to the much-disputed quote by Margaret Thatcher – ‘There is no such thing as Society’). In contrast, the idea of society and thinking of authentic assessment as a socially situated achievement is powerful and transformative. It gives genuine agency to a student’s sense of applying what they learn in higher education to the professional and social roles they later go on to hold, including the member of society they become.

I will illustrate this with two assessment examples. Firstly, from engineering, which is an obvious discipline for authentic assessment in terms of real-world/world of work tasks. An authentic assessment in the conventional sense might be to research and write a report with recommendations on the best affordable and low carbon transport option to get students from the local town to the university or college campus. Implicit in this assessment is that affordable and low carbon are desired attributes for a transport system. There is, however, no explicit sense of why they are desirable and for whom? These could be traits that are prized by the customer and only have an accidental link to the climate emergency and social well-being. Indeed research from McArthur et al., (2021) reveals that students often do not associate an assessment task with its social purposes, even when it seems to explicitly deal with a social need. The first step to make this assessment authentic in my alternative conceptualisation is to make what is implicit and, of social worth, explicit.

If we turn now to English Literature, we can consider the same issues for a non-vocational authentic assessment. Imagine students are doing an assignment on gothic literature, where conventionally they might be asked to write an essay critically exploring a particular novel. Before we even alter the assessment task, we can make this more authentic by explicitly situating it in the social world and issues of social justice. Gothic literature is often used as a device to explore issues of social marginalisation. We can make this explicit link between why we might study gothic literature and social well-being. As I work through the next two principles for rethinking authentic assessment, I will expand on the ways we can develop both these assessment ideas in terms of their authenticity for students’ individual well-being and that of society.

### From task performance to why we value the task

Situating authentic assessment within a social context also helps us to understand why we must think beyond the task performance, which was the feature of so much early work on authentic assessment. Performing a task does not mean an awful lot until we are able to establish that such a task is worth doing. Again, for some, a task may be obviously worth doing if this is what the employer wants. But this reinforces a sense of self-worth purely determined externally rather than intrinsically, whereas the two should inter-relate when we understand the relationship between individual and social well-being.

The climate emergency and the recent COVID-19 pandemic are powerful representations of the interconnectedness of human welfare and well-being. And it is this which, I argue, should be the second pillar of authentic assessment. The reason we need this sense of the value of a task and not the task itself relates to both individual and social well-being, as understood in critical theory. Again, Honneth’s (2004b) concept of esteem recognition, one of the three aspects of his plural theory of social justice, refers to recognising in oneself and being recognised by others, that one has skills, knowledge, or a disposition through which you contribute to wider social well-being. And through this social contribution, their own individual well-being is ensured; thus, individual and social well-being are fundamentally inter-connected in a far more complex way than the traditional liberal view of social well-being as simply the sum-total of individual well-being.

Because we now focus not only on the task but why that task matters, we also transcend traditional associations of authentic assessment only with vocational disciplines. Students in a music conservatoire are participating in authentic tasks because these are tasks that will bring joy to the human world. The usefulness of joy, kindness, and beauty to wider society, and each of us as individuals, provides a powerful, but not purely economic, defence for the need for disciplinary diversity in higher education. Despite what the former UK Secretary of State for Education says, it is never a dead-end degree (Bothwell, 2021) if it helps nurture the sum total of human creativity, diversity, joy, and understanding.

The focus on the reason for the task also challenges our engineering student to rethink conventional ways of calculating costs. Our current system in which we allow industry and businesses to pollute to a reasonable level (within statutory guidelines) and then mitigate this through public funding hides the true cost of production. The reason for the task (climate emergency) shapes the task and hence cannot be separated from it: this has to be part of our understanding of authentic.

There is also something intrinsically important to the individual student of associating their assessment task with the micro detail of people’s lives and not just the ‘big’ issues (because of course the two inter-relate). The engineering student should be able to understand the potential of their work to help children with asthma or poor communities in flood-prone areas: all victims of pollution and the climate emergency. This is the essence of the students’ own esteem recognition and hence their well-being. Individual and social well-being are intwined. Turning to our English Literature student, we could ask her not simply to commentate on how the gothic novel deals with marginalisation, but to think about what that form of marginalisation might feel like. She could write a blog or article on lessons we must learn about marginalisation in society, referencing the novel and demonstrating her engagement with it. Literature is one of the ways in which we either perpetuate myths about our society or explore hidden and unacknowledged forms of oppression. As such understanding literature is an immensely practical act of enormous social value (as well as being enjoyable). In both cases, the authentic assessment experiences require the application of empathy: a sense of what the student does and how this may impact on the lives of others, who the student may never meet and with whom they may have little in common other than shared social membership.

### From the status-quo of real-world/world of work to transforming society

My aim so far has been to rethink the concept of authentic, in the context of assessment, from the task to the reason for the task, and hence the person, and to consider that student/graduate not as an isolated individual but as part of wider society. Authentic assessment should be that which enables a student to find their place in society and be recognised for their contribution to that society. Clearly in many ways, this will happen through their working lives. But we do not have to limit it to that, nor does our concept of work and well-being need to conform only to employer expectations. The relationships between work, well-being, and self-understanding are complex (Guess, 2021).

I return again to Weinstein’s claim that the university serves a unique purpose for society by being separate from that society, enabling an objective and disinterested stance. There is an element of the same thinking in critical theory, but the implications are far from the same. For Adorno, this is about escaping mainstream society, built upon the status-quo, while so clearly a part of that society because otherwise how do you tackle injustice from within a social structure that is itself unjust? Adorno refers to the ease with which people may swim with the current they profess to be so against (Adorno, 2005). Unlike Weinstein, however, Adorno is very much focused on moments of being outwith and within society at the same time, and not an elitist preserve of a select group of intellectuals. Richter (2007) describes this aspect of the Frankfurt School in the idea of ‘paleonomy’ whereby one can stand inside and outside a tradition at the same time, clearly part of that tradition while also breaking from it. Weinstein appears to hope to perpetuate a social order about which he is generally quite satisfied (happy to stay within), while in contrast, critical theory rests of ‘a deep conviction that society is organized unfairly’ (Brookfield, 2003, p. 141). McArthur (2013) draws upon Adorno to argue that in order to achieve greater social justice within and through higher education, it is necessary for higher education to provide moments outside mainstream society: otherwise how does one pursue greater social justice from within an unjust system? This is clearly a very different sense of separation than that implied by Weinstein: with one arguing for the status-quo and the other for radical transformation.

In this final stage, therefore, the concept of authentic assessment becomes not one of joining an existing world *out there* but of being part of the transformation of that world, in all its manifestations within and beyond higher education. Authentic assessment is not assessment that mirrors the world as it is, but that which pushes the possibilities of what the world could be. This is why my understanding of authentic assessment is different to that of Vu and Dall’Alba (2014) even though they may seem superficially alike. We both talk of thinking beyond tasks when it comes to authentic assessment and instead focusing on the student, who they can become and how they live in the world. But the very different theoretical foundations are hugely significant. While making references to the student being in the world, Vu and Dall’Alba’s grounding in Heidegger’s existentialism necessarily means a very different relationship of self and society to that in critical theory. It lacks the sense of inter-relatedness to the point of mutual construction which is inherent in critical theory. Their perspective is inescapably individualistic, because that is the essence of Heidegger’s work.

Another apparent similarity might be that Vu and Dall’Alba critique the task-based understanding of authentic assessment as preparing students for a changing world. Their ontological approach problematises this idea of a changing world. Certainly, much of the more conventional literature on authentic assessment makes reference to this need to prepare for a changing world (Jopp, 2020; Schultz et al., 2021). But we need to stop and consider what we mean by change. Much of this literature speaks to changes in how things will be done in work and/or society and not changes in *why* they should be done. They refer to changes in the ways and means of manufacturing goods, providing services or managing the workforce. The sector in which these changes occur can remain largely unchanged, in that it continues to be based on competitive notions of economic winners and losers, disparities in wealth, and continued high levels of production and consumption. These are changes, adjustments, that perpetuate the status-quo rather than transform it.

Fraser (2003) is helpful here in her distinction between affirmative change and transformational change. Affirmative change relieves some of the consequences of the prevailing system, without seeking to significantly change the system itself. The welfare state is an example of affirmative change, which mitigates the inequalities caused by late capitalism but does not address their causes. Transformative change, in contrast, would focus on causes and work form there. This then is the challenge for authentic assessment. Is it to prepare students for a changing world in the sense of changes in the means of production, the type of industrial base (manufacturing, service, knowledge, financial), and probably a whole lot of new job titles we cannot even imagine? Or is authentic assessment preparing students to change the world, in the sense of contributing to change in which they and others get to live and work in ways that enhance both individual and social well-being?

Our engineering student experiences authentic assessment when it enables her to see that her transformative engagement with disciplinary knowledge, to use Ashwin’s (2020) phrase, can in turn be used to ensure transformative change in society: to not think of the calculations involved in evaluating a transport system solely in isolation, but to see a direct link between them and improving other people’s lives. Our English Literature student’s engagement with gothic literature and marginalisation becomes not simply one of intellectual curiosity (although that is good too) but something that equips her to challenge affirmative adjustments to marginalisation, which are often about bringing the so-called marginalised into the mainstream. She is able to argue for genuine forms of social inclusion which are not based on assumptions of marginal and mainstream.

## Conclusion

These two examples from Engineering and English Literature are only partially developed in this space. I offer them as illustrations of how and why we should think differently about what authentic assessment should mean. They can then be taken into a productive dialogue with the rich field of existing examples of authentic assessment, as outlined recently by Sambell and Brown (2021).

As authentic assessment captures the imagination of more and more academics and higher education leaders, let us ensure it does so through a rich conceptualisation that reaffirms the role of higher education in contributing to greater social justice. There are a number of foundational statements underpinning this article to reinforce: the world of work does matter, but it is part of a larger, complex entity of society; work is often essential to well-being; humans by their nature need to do useful things; such usefulness is not narrow, utilitarian, or purely economic. The concept of ‘real world’ suggests some of us are living in some parallel unreal world. Moreover, its core intention to separate higher education and the real world is deeply troubling. Higher education, and those who study and work within it, is all part of our social world.

Building upon the extensive scholarship on how to go about authentic assessment, I seek to push the boundaries of what makes it authentic. Many of the existing examples of authentic assessment (such as in Sambell & Brown, 2021) remain relevant. But in expanding our sense of why we assess in these authentic ways, the concept of authenticity is itself broadened and made richer. Students, and society, gain more from engagement in these tasks because that engagement has an enriched sense of purpose.

As Adorno tells us, the idea of authenticity is itself susceptible to jargon, buzzwords, and grand claims belying often empty promises. Travel brochures and marketing campaigns are full of promises of authentic experiences, but like the term celebrity, today, this phrase can seem rather devoid of meaning. Our students and our societies deserve a higher education which is committed to individual and social well-being, that is prepared to look beyond the status-quo and which is not fearful of transformative change. Assessment—and particularly authentic assessment—has a vital role to play in this endeavour.
